# Prevalence and Predictors of Postoperative Hypoparathyroidism: A Multicenter Observational Study

**DOI:** 10.3390/jcm14072436

**Published:** 2025-04-03

**Authors:** Reem J. Al Argan, Dania M. Alkhafaji, Shaya Y. AlQahtani, Abdulmohsen H. Al Elq, Feras M. Almajid, Njoud K. Alkhaldi, Zahra A. Al Ghareeb, Moutaz F. Osman, Waleed I. Albaker, Hassan M. Albisher, Yasir A. Elamin, Jamal Y. Al-Saeed, Mohammed H. Al Qambar, Abdulaziz Alwosaibei, Rashid O. Aljawair, Fatima E. Ismaeel, Reem S. AlSulaiman, Jumana G. Al Zayer, Ahmed M. Abu Quren, Jenan E. Obaid, Weeam A. Alhubail, Sarah S. AlThonayan, Mohammed J. Alnuwaysir

**Affiliations:** 1 Department of Internal Medicine, College of Medicine, Imam Abdulrahman Bin Faisal University, Dammam 31441, Eastern Province, Saudi Arabia; dmkhafaji@iau.edu.sa (D.M.A.); saalQahthani@iau.edu.sa (S.Y.A.); wialbakr@iau.edu.sa (W.I.A.); yaelamin@iau.edu.sa (Y.A.E.); drfatimaebrahim@gmail.com (F.E.I.); rsolaiman615@gmail.com (R.S.A.); alzayer.jumana@gmail.com (J.G.A.Z.); 2 Department of Internal Medicine, King Fahad Hospital of the University, Khobar 31952, Eastern Province, Saudi Arabia; 3 Department of Critical Care Medicine, King Fahad Hospital of the University, Khobar 31952, Eastern Province, Saudi Arabia; 4 Department of Surgery, College of Medicine, Imam Abdulrahman Bin Faisal University, Dammam 31441, Eastern Province, Saudi Arabia; fmalmajid@iau.edu.sa (F.M.A.); hmAlbisher@iau.edu.sa (H.M.A.); 5 Department of Surgery, King Fahad Hospital of the University, Khobar 31952, Eastern Province, Saudi Arabia; 6 Department of Internal Medicine, King Fahad Specialist Hospital-Dammam, Dammam 32253, Eastern Province, Saudi Arabia; dr.njoudalkhaldi@gmail.com (N.K.A.); drjamalsaeed@yahoo.com (J.Y.A.-S.); alqambarm@yahoo.com (M.H.A.Q.); aalwosaibei@moh.gov.sa (A.A.); jenan.e.obaid@gmail.com (J.E.O.); weeamah@gmail.com (W.A.A.); 7 Department of Internal Medicine, Qatif Central Hospital, AlQatif 32654, Eastern Province, Saudi Arabia; zaghareeb@gmail.com (Z.A.A.G.); pahmed52@gmail.com (A.M.A.Q.); sara.thonayan@gmail.com (S.S.A.); alnowaysir@gmail.com (M.J.A.); 8 Department of Internal Medicine, King Fahad Military Medical Complex, Dhahran 34313, Eastern Province, Saudi Arabia; moutazosman@hotmail.com (M.F.O.); rshdomr@yahoo.com (R.O.A.)

**Keywords:** hypoparathyroidism, hypocalcemia, lobectomy, total thyroidectomy, transient, permanent, serum calcium, Vitamin D, parathyroid hormone level

## Abstract

**Background/Objectives**: Hypoparathyroidism (HPT) is a common complication following thyroid surgery with an incidence reaching up to 29%, potentially resulting in significant long-term morbidity. To improve its early identification and patient outcomes, we investigated the prevalence and predictors of postoperative HPT. **Methods**: This retrospective, multicenter observational study included patients who underwent thyroid surgery from 2016 to 2022 in four centers located in Saudi Arabia’s Eastern Province. We analyzed demographic data, underlying thyroid or parathyroid conditions, surgical indications, types of procedures, pathology results, and preoperative corrected calcium and vitamin D levels, along with postoperative corrected calcium and parathyroid hormone (PTH) levels. For data analysis, IBM Statistical Package for the Social Sciences (SPSS) Statistics 22 was used, with categorical variables presented as frequencies/percentages and non-normal continuous variables as the median/first quartile (Q1) and third quartile (Q3). Associations were tested with chi-square/Fisher exact tests, medians with Mann–Whitney U-tests, and odds ratios (ORs) with 95% confidence intervals (CIs) via multivariate analysis with statistical significance set at *p* < 0.05. **Results**: A total of 679 cases were included. The median age of patients was 43 years (with 48.9% of them aged 41–60 years), and 82% were female. HPT occurred in 228 cases (35.3%), with 115 (81.0%) experiencing transient HPT and 27 (19.0%) permanent HPT. Multivariate analysis identified total thyroidectomy (OR 2.7, *p* = 0.005), completion thyroidectomy (OR 8.4, *p* = 0.004), and low immediate postoperative PTH level (OR 3.1, *p* < 0.001) as independent predictors of HPT. Central lymph node dissection (CLND; OR 4.03, *p* = 0.004) and low postoperative PTH level (OR 2.56, *p* = 0.049) were significant predictors of permanent HPT. **Conclusions**: Key predictors of HPT include surgical extent and low postoperative PTH level, while CLND and low postoperative PTH level are the strongest predictors of permanent HPT. Careful assessment of these risks when determining the extent of surgery and avoiding unnecessary aggressive procedures can help to minimize the occurrence of HPT. Measuring the PTH level immediately after surgery may aid in identifying high-risk patients for early intervention and appropriate follow-up.

## 1. Introduction

Hypoparathyroidism (HPT) is one of the most prevalent complications after thyroid surgery [1,2]. In the short term, it leads to an increased postoperative hospital stay [3]. Furthermore, it is associated with a need for emergency visits, hospitalization, and the limitation of daily activities in the long term [4], as well as an increased risk of morbidities such as renal impairment and calcification in addition to basal ganglia calcification [5]. According to the existing literature, the incidence of postoperative HPT can reach up to 29% [6], and it may persist and become permanent in up to 5% of cases [7].

The early identification of patients at risk of HPT following thyroid surgery will help in determining those that will benefit most from early treatments, thus improving their outcomes. However, the best approach for the early identification of high-risk patients remains poorly defined. Multiple previous studies have identified various risk factors for the occurrence of postoperative HPT [8,9,10,11,12]; for example, Cho et al. studied predictors of HPT in a total of 1030 patients who underwent total thyroidectomy. The results showed that a relative change in the serum level of calcium of more than 20% on the second postoperative day may be a reliable predictor of post-thyroidectomy HPT [8]. In addition, through multivariate analysis, female gender (*p* = 0.001), the extent of central lymph node dissection (CLND; *p* = 0.017), and the identification of parathyroid gland tissue in permanent pathologic sections were found to be significant predictors of hypocalcemia [8]. Moreover, the postoperative parathyroid hormone (PTH) level has been shown in multiple studies to be an indicator of the development of HPT following thyroid surgery [9,10,11,12]. For instance, Ru et al. studied 537 patients who had a total thyroidectomy. The primary risk variables for long-term postoperative HPT were lymph node dissection (OR 1.594, *p* = 0.011), maximum thyroid diameter (OR 1.254, *p* = 0.032), and PTH level on the first postoperative day (OR 1.199, *p* = 0.001) [10].

Given the inconsistencies in the previously published evidence regarding the prevalence and predictors of HPT following thyroid surgery, we conducted this research. We aimed to investigate the prevalence of HPT and identify its clinical, biochemical, and pathological predictors. These findings could facilitate the early identification of at-risk patients, minimize the need for frequent biochemical testing, shorten hospital stays, and ultimately improve patient outcomes.

## 2. Materials and Methods

### 2.1. Study Design

This is a retrospective multicenter observational study that evaluated all patients who underwent thyroid surgery between January 2016 and December 2022. The study was conducted in four centers in Saudi Arabia’s Eastern province; namely, King Fahad Hospital of the University (KFHU), King Fahad Specialist Hospital-Dammam (KFSH-D), Qatif Central Hospital (QCH), and King Fahad Military Medical Complex (KFMMC).

Inclusion Criteria: All patients aged ≥ 18 years who underwent total thyroidectomy or lobectomy during the period from January 2016 to December 2022 in four centers from the Eastern Province of Saudi Arabia. 

Exclusion Criteria: Patients less than 18 years old and any patients who had thyroid surgery before 2016 or after 2022.

### 2.2. Data Collection Procedure

After obtaining approvals from the institutional review boards of the participating centers, the data of the included patients were extracted from the electronic health record systems of the four centers participating in the study. The following data were collected for the eligible patients:

1. Demographic data (age and sex).
2. Underlying thyroid hormonal status and parathyroid diseases.
3. Indication of surgery (benign or malignant indications).
4. Type of surgery (lobectomy, total thyroidectomy, or completion thyroidectomy with or without lymph node dissection).
5. Postoperative pathology (benign, malignant, or premalignant). Then, postoperative differentiated thyroid cancer (DTC) cases were classified based on the 2015 American Thyroid Association (ATA) risk stratification of thyroid cancer [13].
6. Biochemical and hormonal profile:
  - Preoperative vitamin D level: normal vitamin D level defined as over 75 nmol/L.
  - Pre- and postoperative corrected calcium for albumin levels: normal corrected calcium level defined as 2.1–2.55 mmol/L.
    The following formula was used for the calculation of the corrected calcium level:
    Corrected calcium (mmol/L) = measured total calcium (mmol/L) + 0.02 [40—serum albumin (g/L)]
  - Immediate postoperative PTH level: The first PTH measurement taken within 24 h after surgery. Due to the variations in normal ranges across the four centers, PTH levels were standardized to pmol/L. The median (first quartile–third quartile; Q1–Q3) reference range was 4.25 (1.55–7.24) pmol/L. A low PTH level was defined as <1.5 pmol/L.
  - Follow-up of PTH levels for at least 6 months post-surgery to assess recovery or the persistence of HPT.
7. According to the corrected calcium and PTH levels, patients were categorized into two groups: (1) No hypoparathyroidism, defined as normal corrected calcium and PTH level post-surgery, or (2) hypoparathyroidism, defined as low corrected calcium level with either inappropriately low or undetectable PTH level postoperatively [14]. Thereafter, the cases of HPT were categorized into two groups based on the recovery or persistence of HPT: (1) Transient HPT, defined as recovery of PTH level with stopping replacement therapy at or before 6 months post-surgery, or (2) permanent hypoparathyroidism, defined as the persistence of low PTH level and the continued need for replacement therapy at more than 6 months post-surgery [14].

### 2.3. Statistical Analysis

The data analysis was performed using the IBM Statistical Package for the Social Sciences (SPSS Statistics 22). Categorical variables are presented as frequencies and percentages, while continuous variables are presented as the median and Q1–Q3 for the data that do not follow a normal distribution. To assess the associations between the variables, we used the chi-square or Fisher exact probability test, while, to compare the medians, we used the Mann–Whitney U test. Odds ratios (ORs) and 95% confidence intervals (CIs) were determined via multivariate analysis. Significance was considered at a *p*-value < 0.05.

## 3. Results

### 3.1. Population Demographics and Comorbidities

A total of 679 cases were included. The median age was 43 years with a range of 35–52 years, where 48.9% of our cohort was aged 41–60 years. Females constituted 82% of the total cohort. Most of our patients were euthyroid (in 85.9% of the cases), while only 7.5% and 6.6% had hyper- and hypothyroidism, respectively. Primary hyperparathyroidism was present in 17 cases (2.5%); see Table 1.

### 3.2. Surgery Details and Postoperative Pathology

Surgery was performed in most of our patients for benign multi-nodular goiter (MNG) in 37.1%, followed by thyroid nodules with Bethesda V–VI (32.1%) and Bethesda III–IV (29.7%). Most of our cohort underwent total thyroidectomy (69.5% of the cases), followed by lobectomy (29.2%). CLND was performed in 14.6%, while lateral lymph node dissection (LLND) was performed in 11.6%. Postoperative pathology showed malignancy in 54.2%, while benign pathology was found in 43.7%. 

The most common malignancy in our cohort was papillary thyroid cancer (PTC), in 93.8% of the cases, while follicular thyroid cancer (FTC) was found in 3.8%. According to the ATA risk stratification, 63.8%, 28.7%, and 7.5% of DTC cases had low, intermediate, and high risk, respectively (Table 2).

### 3.3. Baseline Bone Profile and Outcome of HPT

Vitamin D level was measured preoperatively in 405 cases (59.6%), and vitamin D deficiency was observed in 263 cases (38.7%). HPT was found in 228 cases (35.3%) of the total cohort with the available postoperative calcium and PTH data. 

**Figure 1:**
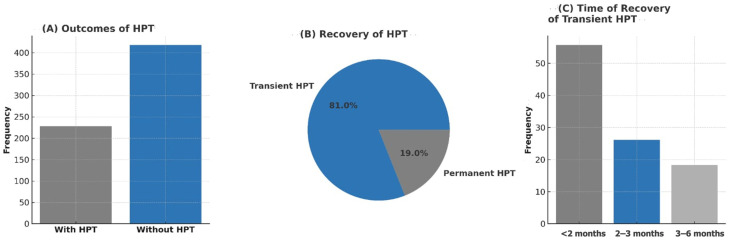
Hypoparathyroidism outcomes. Abbreviations: HPT: Hypoparathyroidism. Figure (**A**): Bar chart showing outcomes of hypoparathyroidism (HPT): frequencies of cases with and without HPT. Figure (**B**): Pie chart showing recovery of HPT: distribution of transient and permanent HPT. Figure (**C**): Bar chart showing time of recovery for transient HPT: the duration of recovery in affected individuals.

Of the 142 cases with available follow-up data for at least 6 months post-surgery, 115 cases (81.0%) had transient HPT, while 27 patients (19.0%) had permanent HPT. Of the transient HPT cases, 64 cases (55.7%) had resolved HPT in <2 months, while 30 cases (26.1%) and 21 cases (18.3%) had resolved HPT in 2–3 and 3–6 months, respectively (Table 3, Figure 1).

### 3.4. Biochemical Parameters of Patients with and Without HPT

**Figure 2:**
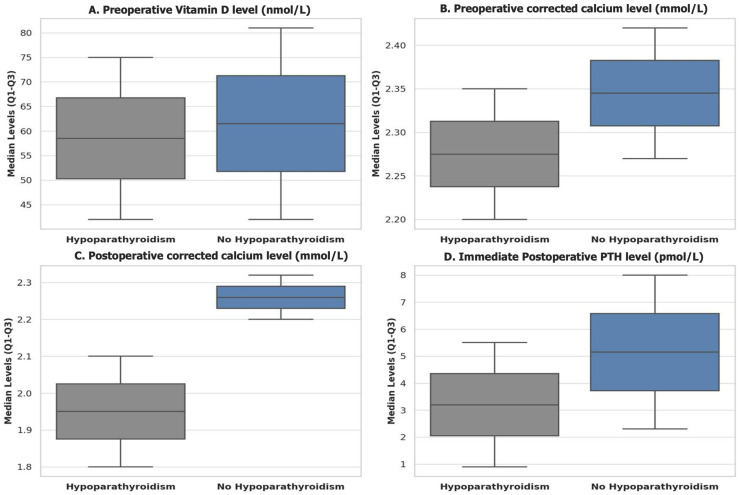
Boxplots comparing biochemical parameters between patients with and without postoperative hypoparathyroidism. Abbreviations: Q1: first quartile; Q3: third quartile; PTH: parathyroid hormone. Figure (**A**): Preoperative vitamin D level (nmol/L): patients without hypoparathyroidism (HPT) had slightly higher median vitamin D levels than those with HPT. Figure (**B**): Preoperative corrected calcium level (mmol/L): patients without HPT showed higher corrected calcium level preoperatively, compared to those who developed HPT. Figure (**C**): Immediate postoperative corrected calcium level (mmol/L): a significant decrease in the corrected calcium level is observed in patients with HPT, compared to those without HPT. Figure (**D**): Immediate postoperative parathyroid hormone (PTH) level (pmol/L): PTH level was significantly lower in patients who developed HPT postoperatively.

Our cohort had a median vitamin D level of 59.4 nmol/L, with a range of 42.2–79.7 nmol/L. Preoperative vitamin D levels were slightly higher in patients without HPT in comparison to those with HPT, with median levels of 60 nmol/L and 56 nmol/L, respectively; however, this difference did not reach statistical significance (*p* = 0.3). On the other hand, preoperative corrected calcium levels were significantly lower in the HPT group (median: 2.28 mmol/L) compared to those without HPT (median: 2.35 mmol/L, *p* = 0.001). This trend continued postoperatively, with the immediate postoperative corrected calcium level being markedly lower in the HPT group (median: 1.97 mmol/L) versus the non-HPT group (median: 2.25 mmol/L, *p* = 0.001). Similarly, the immediate postoperative PTH level was significantly lower in patients with HPT (median: 2.9 pmol/L) when compared to those without HPT (median: 4.8 pmol/L, *p* = 0.001); see Table 4 and Figure 2.

### 3.5. Predictors of Hypoparathyroidism

Univariate analysis was conducted to test for predictors of HPT. We found that age, sex, underlying thyroid hormonal status, and indications of surgery did not have associations with HPT. However, the type of surgery was a predictor of HPT outcome, where total thyroidectomy and completion thyroidectomy were associated with HPT in 81.1% (*p* < 0.001) and 2.6% (*p* = 0.04), respectively. On the other hand, lobectomy was associated with the absence of HPT in 32.5% (*p* < 0.001). Next, the results of final pathology and the type of malignancy were not associated with HPT, except for non-invasive thyroid neoplasm with papillary-like nuclear features (NIFTP), which showed statistical significance for absence of HPT in 3.1% (*p* = 0.03). On the other hand, ATA risk categories of thyroid cancer recurrence showed a statistical association with HPT, where the intermediate risk of thyroid cancer recurrence was associated with HPT in 39.4% (*p* = 0.002) and low-risk thyroid cancer was associated with the absence of HPT in 70.3% (*p* < 0.001). Furthermore, we found a statistically significant association between immediate postoperative PTH level and the likelihood of developing HPT (*p* < 0.001). HPT occurred in 35.5% of the patients with normal immediate postoperative PTH levels, while 45% maintained normal parathyroid function. Conversely, among those with low postoperative PTH level, 19.3% experienced HPT, whereas only 7.9% maintained normal parathyroid function (Table 5). 

Next, we conducted a multivariate analysis for the predictors of HPT, which revealed interesting findings. First, the type of surgery was a predictor of HPT. We found that total and completion thyroidectomies increased the odds of HPT (OR 2.7, *p* = 0.005 and OR 8.4, *p* = 0.004, respectively). However, lobectomy was a protective factor against HPT, with an OR of 0.4 (*p* < 0.0001). Next, the final pathology result was not statistically significant as a predictor of HPT, nor were the ATA categories of thyroid cancer risk of recurrence. On the other hand, low immediate postoperative PTH level was a strong predictor of HPT with an OR of 3.1 (*p* < 0.001); see Table 6.

### 3.6. Predictors of Permanent Hypoparathyroidism

A univariate analysis of predictors for transient versus permanent HPT following thyroidectomy is presented in Table 7. Several factors were analyzed, including demographic characteristics, surgical details, pathology, and biochemical markers. We found that the presence of hypothyroidism was associated with a higher frequency of permanent HPT in 14.8% vs. 4.3% of transient HPT cases (*p* = 0.044). In addition, certain surgical factors, such as CLND, demonstrated statistically significant relationships (*p* = 0.03) with permanent HPT in comparison with transient HPT (37% vs. 13%). Furthermore, a statistically significant association was found between the immediate postoperative PTH level and the risk of transient or permanent HPT (*p* = 0.015). Patients with normal PTH level were more likely to develop transient HPT (69.3%), rather than permanent HPT (37.0%). Conversely, those with a low PTH level had a higher likelihood of permanent HPT (51.9%) compared to transient HPT (30.7%). This suggests that a low PTH level is likely associated with permanent HPT. The other listed factors in Table 7 did not show any significant associations. 

Next, a multivariate analysis indicated that CLND (OR 4.03, *p* = 0.004) and low immediate postoperative PTH level (OR 2.56, *p* = 0.049) were independent predictors of permanent HPT. However, despite the fact that hypothyroidism had a high odds ratio for permanent HPT (3.96), the *p*-value did not reach statistical significance (Table 8).

## 4. Discussion

This is a retrospective multicenter observational study including 679 cases who underwent thyroid surgery at four centers in the Eastern Province of Saudi Arabia in the period from 2016 to 2022. The study aimed to investigate the prevalence and predictors of HPT following thyroid surgery. 

We found several interesting results. First, we identified the prevalence of HPT to be 35.3%. Previous evidence has shown a variable rate of HPT, ranging between 10.3 and 28.8% [15,16,17,18]. We found that 81.0% of our HPT patients had transient HPT, while 19.0% had permanent HPT. Previous evidence reported by Wang et al. revealed different percentages, where, of 637 patients who developed HPT, 333 patients (52.3%) developed transient HPT, leading to a higher percentage of permanent HPT (304 patients, 47.7%) in their cohort [17]. Another study, published by Zou et al., reported transient HPT in 36.1% and long-term HPT in 3.9% of their cases [10].

Next, vitamin D level assessment was performed as part of the preoperative evaluation in 59.6% of the cases, and vitamin D deficiency was found to be prevalent in 38.7%. However, our analysis did not indicate vitamin D deficiency as a predictor of HPT. Similarly, in a study of 361 patients, Manzini et al. have reported that vitamin D deficiency was neither a predictor of hypocalcemia nor a protective factor against permanent HPT [19]. Moreover, Cherian et al. studied 150 post-total thyroidectomy patients and reported a prevalence of vitamin D deficiency of 53.3%. In addition, vitamin D deficiency did not increase the risk of post-thyroidectomy hypocalcemia (*p* = 0.23) [20]. In contrast, a retrospective study by Al-Khatib et al. revealed severe preoperative vitamin D deficiency as a significant independent predictor of postoperative hypocalcemia (OR 7.3; *p* = 0.001) [21]. Other studies have reported a protective effect of vitamin D deficiency against the risk of HPT; for example, in a prospective observational study of 100 patients who underwent total thyroidectomy, individuals with vitamin D deficiency exhibited a lower rate of transient HPT, higher median PTH levels 24 h after surgery, and a smaller decline in postoperative PTH levels when compared to those without vitamin D deficiency [22].

In addition, the extent of surgery was associated with HPT. The univariate analysis results demonstrated that total thyroidectomy and completion thyroidectomy were associated with HPT in 81.1% (*p* < 0.001) and 2.6% (*p* = 0.04) of cases, respectively. However, lobectomy was associated with the absence of HPT (32.5%, *p* < 0.001). These findings were reflected in the multivariate analysis, where total thyroidectomy (OR 2.7, *p* = 0.005) and completion thyroidectomy (OR 8.4, *p* = 0.004) were significant predictors of HPT. On the other hand, lobectomy remained a protective factor against HPT (OR 0.4, *p* < 0.0001). Similarly, a comprehensive meta-analysis encompassing 50,445 patients found that total thyroidectomy was associated with a higher incidence of postoperative complications—including HPT—than thyroid lobectomy [23]. In addition, total thyroidectomy was associated with a higher risk of temporary and permanent hypocalcemia, with a relative risk (RR) of 10.67 (95% CI: 5.75–19.31) and 3.17 (95% CI: 1.72–5.83), respectively [23]. Moreover, another study in 162 patients who underwent thyroid surgery reported the extent of surgery as a risk factor for both transient and permanent HPT [7].

Next, immediate postoperative PTH levels showed a significant association with HPT. The median PTH level was significantly lower in patients with HPT (2.9 pmol/L), compared to that (4.8 pmol/L) in patients without HPT (*p* = 0.001). In addition, univariate analysis indicated a normal immediate postoperative PTH level to be a protective factor against HPT (45%, *p* < 0.001). The multivariate analysis confirmed this finding, demonstrating low immediate postoperative PTH level to be significantly associated with an increased risk of HPT (OR 3.1, *p* < 0.001). Likewise, Grodski et al. studied 76 patients who underwent total thyroidectomy and monitored their PTH levels between 4 and 12 h after surgery, concluding that a single PTH level measurement at this time point accurately predicts those patients who are at risk of hypocalcemia [24]. Moreover, the amount of PTH reduction from baseline can be used to predict HPT. Loncar et al. investigated the use of the postoperative PTH level as a predictor of HPT in 110 patients. They showed that a decrease in the PTH level of more than 70% on the first day after total thyroidectomy is a significant predictor for the development of HPT [12]. Additionally, we found that a low immediate postoperative PTH level is not only a predictor of HPT, but also a significant predictor of permanent HPT, as low PTH levels occurred in 51.9% of permanent HPT cases (*p* = 0.015). In addition, a low immediate postoperative PTH level was an independent predictor of permanent HPT in the multivariate analysis (OR 2.56, *p* = 0.049). Similar results have been reported in a study of 352 patients who underwent total thyroidectomy (alone or with lymph node dissection). Transient HPT was less prevalent in patients who underwent parathyroid auto-transplantation, while patients who had a serum PTH level ≤ 5.95 pmol/L at 4–6 h after surgery had a greater risk of developing permanent HPT (OR 134.84, 95% CI 17.25–1053.82) [3]. Furthermore, another study identified transient hypocalcemia as the most common early complication following total thyroidectomy (49%), while permanent HPT was the most frequent long-term complication (6%). The study also found that an intact parathyroid hormone (iPTH) level on the first postoperative day was a strong predictor of permanent HPT. As a cut-off iPTH level of 5 pg/mL demonstrated 95% sensitivity and 99.6% negative predictive value, the authors concluded that iPTH level on the first postoperative day is a reliable predictor of permanent HPT due to its high negative predictive value, with levels above 5 pg/mL effectively ruling out the presence of permanent HPT [25].

Interestingly, although a low preoperative calcium level did not present a significant association with HPT in the univariate analysis, patients who developed HPT had lower median preoperative calcium level (2.28 mmol/L) compared to those without HPT (2.35 mmol/L), with a statistically significant difference (*p* = 0.001). This suggests that, even within the normal range, a lower preoperative calcium level may be associated with a higher risk of postoperative HPT. Likewise, a retrospective study involving 242 patients with differentiated thyroid cancer who underwent thyroidectomy found that a preoperative calcium level below 2.3 mmol/L was an independent predictor of postoperative hypocalcemia, increasing the risk by 1.7 times [26]. Similar results have been reported in other studies [27,28], further supporting the potential link between lower preoperative calcium levels and an elevated risk of postoperative HPT.

Our analysis did not reveal a significant association between the type of final pathology or malignancy and the risk of HPT, except for NIFTP. Notably, NIFTP appeared to be a protective factor against HPT, as only 0.4% of HPT cases had NIFTP compared to 3.1% of cases without HPT (*p* = 0.03). We did not find any previous studies that have examined the risk of postoperative complications in relation to NIFTP. However, a lower risk of complications is expected, as lobectomy—which is typically recommended for NIFTP when surgery is indicated [29]—carries a lower risk of postoperative complications. We believe that the risk of HPT associated with final pathology or thyroid cancer type is often indirect, primarily influenced by the extent of surgery required; for instance, a retrospective analysis of 401 patients who underwent thyroid surgery reported an 8.5% prevalence of permanent HPT, with a significantly higher incidence following total thyroidectomy (20.2%) compared to near-total thyroidectomy (6.7%) or subtotal thyroidectomy (4.2%) (*p* < 0.0001) [30]. Additionally, a multivariate regression analysis in the same study identified primary total thyroidectomy as a significant risk factor for permanent HPT (OR 6.5; 95% CI: 2.9–14.4; *p* < 0.0001) [30]. This evidence suggests that, while the type of thyroid cancer itself does not inherently cause HPT, the surgical approaches tailored to specific malignancies—such as more aggressive dissection being required for medullary or extensive papillary thyroid carcinomas—increases the risk of this complication. For example, Coimbra et al. have reported a higher incidence of HPT following thyroidectomy in patients with a histopathologic diagnosis of malignancy [7]. Similarly, Xue et al. observed a 49.5% incidence of postoperative HPT in patients with PTC [31].

Moreover, the ATA risk categories of thyroid cancer recurrence showed a statistical association with HPT, where the intermediate risk of thyroid cancer recurrence was associated with HPT in 39.4% (*p* = 0.002) and low-risk thyroid cancer was associated with the absence of HPT in 70.3% (*p* < 0.001). Similarly, a population-based study of 27,912 patients who underwent surgery for differentiated or medullary thyroid cancer was studied for thyroid-surgery-related complications. In multivariable analyses, thyroid-surgery-specific complications were significantly higher in older individuals, patients with comorbidities, and those with either regional (OR, 1.31; 95% CI, 1.19–1.45) or distant disease (OR, 1.85; 95% CI, 1.54–2.21) [32]. In addition, the same study by Xue et al. that was mentioned above examined risk factors of postoperative HPT in patients who underwent surgery for PTC. A univariate analysis demonstrated that tumor size (*p* = 0.034), extra-glandular invasion (*p* = 0.028), bilateral tumors (*p* = 0.045), and bilateral CLND (*p* = 0.028) were significant risk factors of HPT [31]. In addition, a multivariate analysis showed that the independent significant risk factors of postoperative HPT were extra-glandular invasion (OR, 19.30; 95% CI, 2.67–139.67; *p* = 0.003) and bilateral CLND (OR, 1.86; 95% CI, 1.38–9.06; *p* = 0.044) [31]. These studies highlighted that the increased surgical extent in malignant cases is a key factor contributing to the risk of HPT. Moreover, a meta-analysis by Ning et al. demonstrated that the highest risk of HPT occurred in female thyroid cancer patients with lymph node metastasis undergoing total thyroidectomy combined with neck dissection. They concluded that the key to preventing postoperative HPT lies in the selection of an optimal surgical approach and intraoperative protection [33]. Their findings support the idea that more extensive surgical procedures—which are more frequently needed in malignant thyroid conditions, especially when lymph node dissection is involved—are associated with a higher risk of injuring or inadvertently removing the parathyroid glands.

In addition to low immediate postoperative PTH level as a predictor of permanent HPT, we found that CLND was also statistically significant (37%, *p* = 0.03) in the univariate analysis and remained an independent predictor in the multivariate analysis (OR 4.03, *p* = 0.004). Previous studies have shown that individuals who undergo total thyroidectomy with lymph node dissection have a higher incidence of HPT [31,33]. However, whether CLND contributes to transient or permanent HPT remains controversial. Some studies have reported an association between total thyroidectomy with CLND and transient (but not permanent) HPT [34]. Conversely, other studies have linked this surgical procedure to permanent HPT as well [35,36]. For example, a meta-analysis by Zhao et al. found a significantly higher rate of both temporary (OR 2.28, 95% CI: 1.92–2.27) and permanent (OR 1.84, 95% CI: 1.15–2.95) HPT in patients who underwent total thyroidectomy with CLND, compared to those who had total thyroidectomy alone [35]. The most likely cause of permanent HPT following CLND is the inadvertent removal of or injury to the parathyroid glands. Due to their anatomical location, the lower parathyroid glands are particularly at risk during CLND [37]. Consistent with previous studies, our findings demonstrated a significant association between CLND and the development of permanent HPT. Therefore, our findings support the recommendation of the careful selection of the best surgical approach and avoiding unnecessary aggressive procedures in order to prevent HPT.

Furthermore, hypothyroidism was found to have a significant association with permanent HPT in the univariate analysis (14.8%, *p* = 0.044) but did not reach statistical significance in the multivariate analysis (OR 3.96, *p* = 0.05). A previous meta-analysis by Gan et al. reported Hashimoto’s thyroiditis—which is a common cause of hypothyroidism—to have an increased risk of transient hypocalcemia compared with benign nodules (16.85% vs. 13.20%, *p* < 0.001). However, their analysis did not indicate a significant difference in terms of the risk for permanent HPT [38].

## 5. Conclusions

Hypoparathyroidism (HPT) is a common complication after thyroid surgery, the most significant predictors of which were found to be the extent of surgery and a low immediate postoperative PTH level. In addition, CLND and a low immediate postoperative PTH level were indicated as independent predictors of permanent HPT. 

Surgeons should minimize the extent of surgery, when possible, especially avoiding unnecessary CLND unless it is indicated. Furthermore, postoperative PTH level monitoring is crucial for the early identification of at-risk patients, and early intervention is warranted for this patient group. Long-term follow-up is necessary for those with persistently low PTH level in order to ensure appropriate lifelong management in cases of permanent HPT. 

We advocate for long-term prospective data in order to better understand the predictors of postoperative hypoparathyroidism and its impacts on patient morbidity. Such data would be instrumental in guiding optimal approaches for managing these cases in the future.

## 6. Strengths and Limitations

The primary strength of our study lies in its robust sample size and the inclusion of data collected from multiple centers. This approach enhances the representativeness of the findings and provides results with higher generalizability. However, the study is not without limitations, the most significant being its retrospective design. While our findings offer valuable insights, we recognize the need for prospective long-term studies to gain deeper insights into the predictors of postoperative hypoparathyroidism, aiding in its optimal care and management. 

## Figures and Tables

**Table 1:** Demographics of study population.

		Frequency	Percentage
Age (years) Median (Q1–Q3) 43 (35–52)	<20	12	1.8
	20–40	265	39.0
	41–60	332	48.9
	61–80	70	10.3
Sex	Male	122	18.0
	Female	557	82.0
Underlying thyroid hormonal status	Hyperthyroidism	51	7.5
	Hypothyroidism	45	6.6
	Euthyroid	583	85.9
Underlying parathyroid disease	Primary hyperparathyroidism	17	2.5

Abbreviations: Q1: first quartile; Q3: third quartile.

**Table 2:** Surgery details and postoperative pathology.

		Frequency	Percentage
Indication of surgery	Grave’s Disease	7	1.0
	Bethesda V–VI	218	32.1
	Bethesda III–IV	202	29.7
	Benign multi-nodular goiter	252	37.1
Type of surgery	Total thyroidectomy	472	69.5
	Completion thyroidectomy	9	1.3
	Lobectomy	198	29.2
Neck dissection	Central lymph node dissection	99	14.6
	Lateral lymph node dissection	79	11.6
Pathology	Malignant	368	54.2
	Benign	297	43.7
	Premalignant (NIFTP)	14	2.1
* Type of malignancy	Papillary thyroid cancer	345	93.8
	Follicular thyroid cancer	14	3.8
	Medullary thyroid cancer	2	0.5
	Anaplastic thyroid cancer	1	0.3
	Poorly differentiated thyroid carcinoma	4	1.1
	Lymphoma	2	0.5
^†^ ATA risk classification of DTC (PTC and FTC) cases	Low	229	63.8
	Intermediate	103	28.7
	High	27	7.5

Abbreviations: NIFTP: non-invasive thyroid neoplasm with papillary-like nuclear features; ATA: American Thyroid Association; DTC: differentiated thyroid cancer; PTC: papillary thyroid cancer; FTC: follicular thyroid cancer. * The percentages were calculated out of malignant cases (total: 368 cases). ^†^ The percentages were calculated out of DTC cases (total: 359 cases).

**Table 3:** Hypoparathyroidism outcomes.

	Frequency	Percentage
* Hypoparathyroidism		
Cases with hypoparathyroidism	228	35.3
Cases without hypoparathyroidism	418	64.7
^†^ Recovery of hypoparathyroidism		
Transient hypoparathyroidism	115	81.0
Permanent hypoparathyroidism	27	19.0
Time of recovery of transient hypoparathyroidism		
<2 months	64	55.7
2–3 months	30	26.1
3–6 months	21	18.3

* The percentages were calculated out of the total cases with available postoperative serum calcium and parathyroid hormone (PTH) levels (total: 646 cases). ^†^ The percentages were calculated out of the total cases with available follow-up data for serum PTH level for at least 6 months (total: 142 cases).

**Table 4:** Biochemical parameters of patients with and without hypoparathyroidism.

	Total Median (Q1–Q3)	Hypoparathyroidism Median (Q1–Q3)	No Hypoparathyroidism Median (Q1–Q3)	*p*-Value
Preoperative vitamin D level (nmol/L)	59.4 (42.2–79.7)	56 (42–75)	60 (42–81)	0.3
Immediate preoperaive corrected calcium level (mmol/L)	2.3 (2.23–2.4)	2.28 (2.2–2.35)	2.35 (2.27–2.42)	0.001
Immediate postoperaive corrected calcium level (mmol/L)	2.2 (2.05–2.3)	1.97 (1.8–2.1)	2.25 (2.2–2.32)	0.001
Immediate postoperative PTH level (pmol/L)	4.14 (2–7.2)	2.9 (0.9–5.5)	4.8 (2.3–8)	0.001

Abbreviations: Q1: first quartile; Q3: third quartile; PTH: parathyroid hormone.

**Table 5:** Predictors of post-thyroidectomy hypoparathyroidism (univariate analysis).

Factor	Hypoparathyroidism		*p*-Value
	Yes Frequency (Percentage)	No Frequency (Percentage)	
Age: Median (Q1–Q3) (Years)	42 (35–50)	44 (35–53)	0.17
Sex			
Male	38 (16.7%)	81 (19.4%)	0.381
Female	190 (83.3%)	335 (80.1%)	
Underlying comorbidities			
Hyperthyroidism	16 (7%)	33 (7.9%)	0.68
Hypothyroidism	18 (7.9%)	25 (6%)	0.35
Euthyroid	194 (85.1%)	358 (86.1%)	0.72
Underlying parathyroid disease	5 (2.2%)	12 (2.9%)	0.6
Indication of surgery			
Grave’s disease	2 (0.9%)	5 (1.2%)	0.8
Bethesda V–VI	82 (36%)	129 (31%)	0.14
Bethesda III–IV	58 (25.4%)	131 (31.3%)	0.07
Multi-nodular goiter	86 (37.7%)	151 (36.1%)	0.7
Type of surgery			
Total thyroidectomy	185 (81.1%)	278 (66.5%)	<0.001
Completion thyroidectomy	6 (2.6%)	2 (0.5%)	0.04
Lobectomy	37 (16.2%)	136 (32.5%)	<0.001
Central lymph node dissection			
Yes	40 (17.5%)	56 (13.4%)	0.146
No	188 (82.5%)	360 (86.1%)	
Lateral lymph node dissection			
Yes	25 (11%)	53 (12.7%)	0.51
No	203 (89%)	363 (86.8%)	
Pathology			
Benign	95 (41.7%)	177 (42.3%)	0.83
Malignant	131 (57.5%)	227 (54.3%)	0.34
Premalignant (NIFTP)	1 (0.4%)	13 (3.1%)	0.03
Type of malignancy			
Papillary thyroid cancer	123 (53.9%)	213 (51%)	0.56
Follicular thyroid cancer	4 (1.8%)	9 (2.2%)	0.66
Medullary thyroid cancer	0 (0%)	2 (0.5%)	0.30
Anaplastic thyroid cancer	0 (0%)	1 (0.24%)	0.45
Poorly differentiated thyroid carcinoma	2 (0.9%)	2 (0.5%)	0.54
Lymphoma	2 (0.9%)	0 (0%)	0.05
ATA risk classification of differentiated thyroid cancer cases			
Low risk	65 (51.2%)	156 (70.3%)	<0.001
Intermediate risk	50 (39.4%)	52 (23.4%)	0.002
High risk	12 (9.4%)	14 (6.3%)	0.29
Preoperative vitamin D level			
Normal	41 (18%)	86 (20.6%)	0.10
Low	98 (43%)	158 (38%)	
Preoperative corrected calcium level			
Normal	167 (73.2%)	329 (78.7%)	0.107
Low	4 (1.8%)	3 (0.7%)	
Immediate postoperative PTH level			
Normal	81 (35.5%)	187 (45%)	<0.001
Low	44 (19.3%)	33 (7.9%)	
Immediate postoperative corrected calcium level			
Normal	0	418 (100%)	NA
Low	228 (100%)	0	

Abbreviations: Q1: first quartile; Q3: third quartile; NIFTP: non-invasive thyroid neoplasm with papillary-like nuclear features; ATA: American Thyroid Association; PTH: parathyroid hormone; NA: non-applicable.

**Table 6:** Predictors of post-thyroidectomy hypoparathyroidism (multivariate analysis).

Factor	Hypoparathyroidism	
	OR (95% CI)	*p*-Value
Age	0.99 (0.98–1)	0.161
Underlying thyroid hormonal status		
Hyperthyroidism	0.89 (0.5–1.6)	0.7
Hypothyroidism	1.3 (0.7–2.4)	0.377
Type of surgery		
Total thyroidectomy	2.7 (1.8–4.1)	0.005
Completion thyroidectomy	8.4 (2–35)	0.004
Lobectomy	0.4 (0.2–0.5)	<0.0001
Neck dissection		
Central lymph node dissection	1.4 (0.9–2.2)	0.13
Lateral lymph node dissection	0.9 (0.5–1.5)	0.7
Pathology		
Benign	7.2 (0.98–59)	0.057
Malignant	6.2 (0.89–54)	0.061
Premalignant (NIFTP)	0.14 (0.02–1.1)	0.06
ATA risk classification of differentiated thyroid cancer cases		
Low risk	0.5 (0.2–1.1)	0.08
Intermediate risk	1.2 (0.5–2.8)	0.84
High risk	1.5 (0.7–3.4)	0.3
Low immediate postoperative PTH level	3.1 (1.9–5.3)	<0.001

Abbreviations: OR: odds ratio; CI: confidence interval; NIFTP: non-invasive thyroid neoplasm with papillary-like nuclear features; ATA: American Thyroid Association; PTH: parathyroid hormone.

**Table 7:** Predictors of post-thyroidectomy transient vs. permanent hypoparathyroidism (univariate analysis).

Factor	Hypoparathyroidism		
	Transient Frequency (Percentage)	Permanent Frequency (Percentage)	*p*-Value
Age: Median (Q1–Q3) (Years)	42 (33–49)	45 (36–53)	0.378
Sex			
Female	20 (17.4%)	4 (14.8%)	0.748
Male	95 (82.6%)	23 (85.2%)	
Underlying comorbidities			
Hyperthyroidism	6 (5.2%)	2 (7.4%)	0.65
Hypothyroidism	5 (4.3%)	4 (14.8%)	0.044
Euthyroid	104 (90.4%)	21 (77.8%)	0.07
Underlying parathyroid disease	4 (3.5%)	1 (3.7%)	0.9
Indication of surgery			
Grave’s Disease	1 (0.9%)	0 (0%)	0.62
Bethesda V–VI	40 (34.8%)	12 (44.1%)	0.45
Bethesda III–IV	34 (29.6%)	4 (14.8%)	0.21
Multi-nodular goiter	40 (34.8%)	11 (40.7%)	0.78
Type of surgery			
Total thyroidectomy	96 (83.5%)	24 (88.9%)	0.31
Completion thyroidectomy	3 (2.6%)	2 (7.4%)	0.201
Lobectomy	16 (13.9%)	1 (3.7%)	0.16
Central lymph node dissection			
Yes	15 (13%)	10 (37%)	0.03
No	100 (87%)	17 (63%)	
Lateral lymph node dissection			
Yes	18 (15.7%)	2 (7.4%)	0.268
No	97 (84.3%)	25 (92.6%)	
Pathology			
Benign	52 (45.2%)	9 (33.3%)	0.37
Malignant	62 (53.9%)	18 (66.7%)	0.34
Premalignant (NIFTP)	1 (0.9%)	0 (0%)	0.62
Type of malignancy			
Papillary thyroid cancer	59 (51.3%)	18 (66.7%)	0.205
Follicular thyroid cancer	1 (0.9%)	0 (0%)	0.66
Poorly differentiated thyroid cancer	1 (0.9%)	0 (0%)	0.38
Lymphoma	1 (0.9%)	0 (0%)	0.38
ATA risk classification of differentiated thyroid cancer cases			
Low risk	31 (51.7%)	7 (38.9%)	0.274
Intermediate risk	26 (43.3%)	8 (44.4%)	0.97
High risk	3 (5.0%)	3 (16.7%)	0.11
Preoperative vitamin D level			
Normal	25 (21.7%)	8 (29.9%)	0.50
Low	58 (50.4%)	13 (48.1%)	
Preoperative corrected calcium level			
Normal	100 (87%)	22 (81.5%)	0.5
Low	2 (1.7%)	1 (3.7%)	
Immediate postoperative PTH level			
Normal	52 (69.3%)	10 (37.0%)	0.015
Low	23 (30.7%)	14 (51.9%)	
Immediate postoperative corrected calcium level			
Normal	0 (0%)	0 (0%)	NA
Low	115 (100%)	27 (100%)	

Abbreviations: Q1: first quartile; Q3: third quartile; NIFTP: non-invasive thyroid neoplasm with papillary-like nuclear features; ATA: American Thyroid Association; PTH: parathyroid hormone; NA: non-applicable.

**Table 8:** Predictors of post-thyroidectomy permanent hypoparathyroidism (multivariate analysis).

	Permanent Hypoparathyroidism	
	OR (95% CI)	*p*-Value
Central lymph node dissection	4.03 (1.5–10.5)	0.004
Low immediate postoperative PTH level	2.56 (1.01–6.5)	0.049
Hyperthyroidism	1.7 (0.3–8.7)	0.635
Hypothyroidism	3.96 (1.1–16)	0.05

Abbreviations: OR: odds ratio; CI: confidence interval.

## Data Availability

The data presented in this study are available upon request from the corresponding author.

## Abbreviations

The following abbreviations were used in this manuscript:

HPT	Hypoparathyroidism
PTH	Parathyroid hormone
CLND	Central lymph node dissection
LLND	Lateral lymph node dissection
DTC	Differentiated thyroid cancer
KFHU	King Fahad Hospital of the University
KFSH-D	King Fahad Specialist Hospital-Dammam
KFMMC	King Fahad Military Medical Complex
QCH	Qatif Central Hospital
TSH	Thyroid-stimulating hormone
T4	Thyroxine
T3	Triiodothyronine
ATA	American Thyroid Association
Q1	First quartile
Q3	Third quartile
OR	Odds ratios
CI	Confidence interval
SD	Standard deviation
ETE	Extrathyroidal extension
PTC	Papillary thyroid cancer
FTC	Follicular thyroid cancer
NIFTP	Non-invasive thyroid neoplasm with papillary-like nuclear features
